# Evaluation of an Interactive Web-Based Health Program for Weight Loss—A Randomized Controlled Trial

**DOI:** 10.3390/ijerph192215157

**Published:** 2022-11-17

**Authors:** Urs Alexander Fichtner, Christoph Armbruster, Martina Bischoff, Phillip Maiwald, Matthias Sehlbrede, Iris Tinsel, Judith Brame, Jan Kohl, Daniel König, Reinhard Fuchs, Ramona Wurst, Erik Farin-Glattacker

**Affiliations:** 1 Section of Health Care Research and Rehabilitation Research, Institute of Medical Biometry and Statistics, Faculty of Medicine, Medical Center—University of Freiburg, 79106 Freiburg, Germany; 2 Department of Sport and Sport Science, University of Freiburg, 79117 Freiburg, Germany; 3 Department of Sport Science, Institute for Nutrition, Sports and Health, University of Vienna, 1150 Vienna, Austria; 4 Department of Nutritional Sciences, Institute for Nutrition, Sports and Health, University of Vienna, 1090 Vienna, Austria

**Keywords:** weight loss, online coaching, RCT, weight reduction, behavioral change, digital health, healthy eating, dietary, web platform, personalized health

## Abstract

Personal behavior patterns, such as unhealthy diet and lack of physical activity, lead to the development of overweight and obesity. These are associated with other comorbidities, reduced quality of life, premature frailty and increased mortality. Personalized web-based interventions are promising in inducing behavioral change leading to effective reductions in body weight at relatively low costs. However, the long-term effects have not been thoroughly investigated. This work evaluates the effectiveness of web-based interactive weight loss coaching and compares it to a non-interactive web-based health program. Therefore, a randomized controlled trial (RCT) was implemented, measuring primary and secondary outcomes at four time points (program start; end of the 12-week-program; 6 months after program end, 12 months after program end). The net sample covered 1499 subjects in the intervention group and 1492 in the control group. On average, the IG was 43 years old (±13.6), 80.1% male, and had 86.4 kg body weight (±16.1) at baseline. The CG was 42.7 years old (±13.9), 79.5% male and had a mean body weight of 86.1 (±15.7). Multilevel analyses with fixed effects were carried out both from the perspective of an intention-to-treat (ITT) and a complete cases approach (CCA). In sum, our adjusted models suggest a weight loss of 0.4 kg per time point. At the end of the program, significant differences in weight loss in % to baseline (delta M = 1.8 in the CCA) were observed in favor of the intervention group. From a long-term perspective, no superiority of the intervention group in comparison to the control group could be found. More intensive use of the program was not statistically associated with higher weight loss.

## 1. Introduction

Personal behavior patterns, such as unhealthy diet and lack of physical activity, lead to the development of overweight and obesity [1]. The prevalence increased worldwide since the 1970s to approximately 1.9 billion in 2016 [2]. Especially in Germany, more than half of adults are overweight (54.0%). Gender differences pronounce mostly at a body mass index (BMI) between 25 kg/m^2^ and 30 kg/m^2^, indicating overweight. According to this, the prevalence is 43.3% for men and 28.8% for women, while there are only minimal prevalence differences for obesity (BMI over 30 kg/m^2^) [3]. In addition to a high risk of a variety of chronic diseases such as type 2 diabetes mellitus, cardiovascular diseases, various cancers, musculoskeletal diseases and premature death [2,4,5], overweight and obesity represent a high financial burden for the health care system [6]. Kent et al. showed that annual health care costs are 12% higher for overweight and 36% higher for obese individuals than for those of normal weight [7].

Overweight and obesity have a high public health relevance. For obesity in particular, the goal is to stop a further increase in prevalence [8,9]. Different treatment approaches and preventive strategies (e.g., surgical, pharmacological, nutritional, psychological) usually fail to achieve long-term success [10]. For those with the condition, a profound behavioral change towards a healthy lifestyle is essential [11]. However, a variety of barriers such as a lack of acceptance, motivation and time, as well as chronic conditions, low socio-economic status, or insufficient availability of obesity treatments and professional expertise affect this process [12].

Meanwhile, numerous digital treatment approaches arose in order to achieve long-term weight reduction or maintenance [13,14,15,16]. Web-based interventions in this context can provide many advantages [17]. Compared to traditional face-to-face treatment, web-based approaches are permanently available, lead to lower costs and waiting times and ensure a high degree of anonymity [18,19,20]. A review from 2017 suggests that web-based interventions contribute to positive effects in terms of weight reduction or maintenance and improve lifestyle [15].

Several primary studies can be found, which examined the effects of web-based interventions in overweight and obese adults over various periods [13,14,16,21,22,23]. 

Mehring et al. conducted a randomized controlled trial (RCT) in which overweight adults received an individualized and automated web-based weight loss program as an add-on to their primary care (usual care) [14]. Three additional calls during the intervention phase aimed to motivate and support the participants. The 12-week intervention resulted in significant differences between the intervention group (IG) and control group (CG) in terms of weight loss, waist circumference, and BMI (4.2 kg vs. 1.7 kg; 6.9 cm vs. 2.4 cm; 1.5 kg/m^2^ vs. 0.6 kg/m^2^). However, this study has some limitations regarding randomization: participants in the IG showed significantly younger age at baseline, more weight loss attempts in the past, and worked more often full-time [14].

Beleigoli et al. examined mid-term effects in a three-arm RCT. The two IGs received either the 24-week weight loss program with computer-delivered feedback solely through a web-based platform or with a supplemental 12-week support (private forum) from a dietitian [21]. The CG was provided with non-personalized recommendations regarding diet and physical activity through an e-booklet or videos. After 12 weeks, there were significant differences in weight loss and BMI between the two IGs and the CG (weight loss: 1.14 kg resp. 1.36 kg vs. 0.56 kg; BMI: 0.41 kg/m^2^ resp. 0.5 kg/m^2^ vs. 0.2 kg/m^2^). No significant differences existed between the IGs. After 24 weeks, however, only the IG with a supplemental 12-week support showed significantly greater effects compared to the CG. The finding, that additional intervention features lead to larger effects, is in line with the RCT by Collins et al. [22]. Both the IG and CG received a 24-week web-based weight loss program with additional features in the IG. Both groups showed significant effects after 12 and 24 weeks in terms of weight loss, BMI and waist circumference compared to baseline [22]. The IG showed larger effects at both measurement points, but these lost statistical significance.

Mensorio et al. investigated the effects of overweight people with hypertension in the context of a 12-week web-based weight management program [13]. After the intervention, the IG presented significantly lower BMI and waist circumference compared to the CG (usual care). At 12-month follow-up in the IG, these effects only persisted for waist- and hip circumference compared to baseline. After the CG gained access to the intervention, hip and waist circumference also decreased [13].

In another three-arm RCT, the intervention period took 12 months in total. Both IGs received a free weight loss program, while one of the two groups also received an activity tracker. The CG obtained an impersonal online newsletter with information on physical activity, healthy eating and the benefits of losing weight. After 3 months, the IGs showed a significantly greater weight loss compared to the CG but not between the IGs. After the 12-month intervention, the effects in all three groups converged (CG: 1.2 kg, IG without activity tracker: 2.1 kg, IG with activity tracker: 1.6 kg) [16].

Many of the web-based interventions involve personal contact (e.g., phone or video calls or text messages, resp. chats) with professional trainers or counsellors [14,21,24]. However, this support is often limited to the duration of the program and it remains unclear how sustainable the interventions are afterwards, when personal supervision is withdrawn. Binks and Mierlo argue, that users appear not to use health information optimally in absence of personal contact [23]. Therefore, insights into the sustainability in terms of behavioral change of tailored web-based interventions without personal contact in a real-life setting are needed.

The aim of this randomized controlled trial was to investigate the long-term effectiveness of a fully automated web-based health coach in relation to weight loss in the general adult population. For this, we compared an interactive web-based health coach (IG) with a passive web-based health information platform (CG). We expected a significant and sustainable weight loss (12 months after end of program) in the IG (Hypothesis 1). Furthermore, we assumed that this weight loss is significantly greater in the IG than in the CG (Hypothesis 2). In addition, we hypothesized that more intensive use of the web-based health coach is related to higher decrease in body weight in the long-term perspective (Hypothesis 3). Lastly, to evaluate the clinical relevance of any weight loss found, it is important to identify how many of the subjects can lose and maintain at least 5% of their initial weight [25,26].

## 2. Materials and Methods

### 2.1. Development Context

The weight loss coaching (WLC) is part of a multimodal health program that addresses individual health goals such as increasing fitness and smoking cessation. It was continuously developed since 2003 under the lead of the German Techniker statutory health insurance fund (TK) involving an interdisciplinary team from Medicine, Sports Science and Psychology. While the technical implementation of the web-based health intervention was led by Vilua GmbH, the evaluation of the coach was conceptualized and carried out by the Section of Health Care Research and Rehabilitation Research, Medical Center Freiburg and the Department of Sport and Sport Science—both affiliated to the University of Freiburg.

### 2.2. Evaluation Approach

The evaluation approach is split into a formative and a summative component following the international standard for complex interventions [27]. While the formative evaluation refers to the process of implementation, the usage behavior and compliance of the users, usability and user satisfaction, the summative evaluation focusses on the investigation of short- and long-term effectiveness of the coach in terms of the targeted goal (weight loss, increase in fitness and smoking cessation). This work exclusively reports the summative evaluation of the WLC, while the results of the evaluation of the other coachings and the formative evaluation component will be published elsewhere. Furthermore, the evaluation includes a regional sub-study, examining medical outcomes of the WLC, which is also not part of this paper (Kohl et al., forthcoming). For a schematic overview of the evaluation, see Figure S1 (Supplementary Material).

To investigate effectiveness of health interventions, a randomized controlled trial is considered as gold standard, promising a high level of evidence [28]. Here, different types of analyses are offered, depending on the research goal. The intention-to-treat approach includes all individuals assigned to the group, regardless whether they completed the intervention (here: represented by survey completion) as intended or not [29]. By this, the method represents a relatively strict and unbiased effectiveness testing, since potentially existing low or opposite effects (e.g., induced by dropping out) might be included. The complete cases approach (CCA) (also referred as per-protocol analysis) reduces the analysis sample to only those who accomplished the intervention (here: represented by survey completion) as intended and excludes dropouts and non-compliant users. Thus, this approach offers a nearer approximation towards the potential effectiveness of the intervention; however, it is more far away from a realistic setting, since it artificially assumes the optimal condition that all individuals use the intervention as intended and might result in overestimated effects for real-world settings. Furthermore, the CCA can be considered as sensitivity analysis for missing imputation, which is commonly used in intention-to-treat approaches. Therefore, the combination of both methods is suggested [30]. In this work, we applied primarily intention-to-treat analyses to estimate an effect as realistic as possible and compared them with complete cases analyses as a means of sensitivity analysis.

### 2.3. Study Design

Since the WLC is developed and improved continuously, fixed versions of the programs from the end of December 2019, which were not changed during the observation period, were used for the RCT. All study participants were given access to a health goal-specific, web-based program. Two groups were defined: The IG is characterized by an interactive program with protocols, reactive algorithms, videos, health information, recipe suggestions and push messages. IG participants were recommended to use the WLC for 12 weeks, while individual action planning with adaptable intensity was implemented (weight loss goal of 3 or 5 kg within 12 weeks). The CG was enabled to use the non-interactive information web platform without any personalized feedback. 

The RCT comprises 4 measurement time points: t0 baseline measurement (before randomization), t1 post intervention measurement (12 weeks after program start), t2 follow-up measurement (6 months after end of program) and t3 end of study measurement (12 months after end of program). At each time point, an online questionnaire was used measuring, among others, sociodemographic and basic health information (t0), body weight (all time points, self-administered in kg), subjective general health in relation to others (t0, inspired by the Berlin Risk Appraisal and Health Motivational Study [31]), health impairment by comorbidity (t0, KOMO-Score [32]), mental disorders (all time points, PHQ-2 depression screener [33]) and weight effective and ineffective social support (all time points, inspired by WEISS Questionnaire (Lee et al., in press) which represents a further development of the Social Support for Eating Habits and Social Support for Physical Activity Scales [34]). Furthermore, a follow-up questionnaire was provided to dropouts, surveying reasons for quitting and usage patterns. 

Besides the primary outcome measure, body weight in kg, we also measured secondary outcomes at each time point: physical activity (BSA-F3 [35]), goal intention (modified item [36]), (healthy) eating behavior (FEG [37]), nutrition-related self-efficacy [38], consequence expectation [31], health-related quality of life (SF-12 [39]). For details and assumed relationships, see study protocol [40].

After completing the baseline questionnaire, the online RCT participants were given access to their selected health program. Meanwhile, they were randomized to one of the two RCT groups by permuting block randomization with variable block sizes of 4, 6 and 8 to ensure equal distribution in both groups [40]. Blinding was not possible, since the respondents had to give informed consent to participate in advance. Likewise, blinded data analysis could not be ensured either, since the data collected indicate the study arm affiliation.

### 2.4. Inclusion Criteria

Inclusion criteria were healthy persons of any sex, aged 18 years and older regardless of their health insurance. In case of pre-existing conditions or health impairments, subjects required a medical assessment for suitability in advance. Participation was only possible in one of the three coaches (smoking cessation, weight loss or increasing fitness); at the same time, however, members of the WLC could use modules of the fitness coach as well. Subjects who were participating in another study aiming to change health behavior towards the respective goal were not included. For the WLC, pregnant or breastfeeding women, individuals with a circumference > 200 cm or BMI > 40 kg/m^2^ or current underweight or underweight after a loss of 3 kg were excluded.

### 2.5. Recruitment

In order to recruit individuals for the study, several campaigns were launched using different media. Recruitment phase was from 1 January 2020 to 28 September 2020. The deadline for last patient-in was 5 October 2020. The due date for data collection was 10 January 2022. The recruitment strategy included Google marketing campaigns, communication channels of the Techniker statutory health insurance fund (Website, Newsletter, Twitter, news feeds), local newspapers and radio channels, a German pharmacy magazine, as well as short articles in the magazines “Men’s Health” and “Women’s Health”. Participation was incentivized by a EUR 25 coupon code from Amazon. Due to high dropout rates after t1, the incentivization strategy was changed during the study to EUR 10 coupons per each measurement time point.

Before recruitment started, we executed a sample size calculation assuming an α error of 0.05, an aimed statistical power of 0.8 and an assumed effect size of 0.5 allowing 10% buffer for invalid questionnaires. In sum, we calculated a necessary sample size of 1114 persons equally distributed on the two groups (for details, see [40]).

### 2.6. Methods

For analysis, data were cleaned and prepared according to the coding manuals—if available—provided by the authors of the scales used. Furthermore, missing data had to be imputed by multiple imputation with the two-stage method [41] based on the assumption of a random distribution. In total, we executed 100 imputations.

To investigate H3, user behavior needed to be operationalized. Therefore, latent class analysis was applied to identify systematic usage patterns and user types were categorized (Wurst et al., forthcoming). Standardized effect sizes and standardized response means were applied to estimate the size of effects. To answer the central research questions, multi-level models for change were calculated after a check of suitability (calculating the intra-class coefficient ICC). On level 1, intra-individual changes for each person were considered, on level 2, inter-individual variation in each parameter was modeled and an interaction term of group and time was included. All models were adjusted for the a priori defined covariates subjective health, comorbidity impairments, mental disorders and social support [40]. For examination of H3, only the IG was used and an interaction term of user type and outcome change over time was modeled. For all multi-level models, fixed effects were estimated. We applied the same models for both the primary outcomes and the secondary outcomes. All analyses were carried out in R statistics (Version 4.1.3) and R Studio (Version 2021.09.1) software using the packages mice, lme4, nlme, robustlmm, micemd and mitml. 

## 3. Results

### 3.1. Sample Size

**Figure 1:**
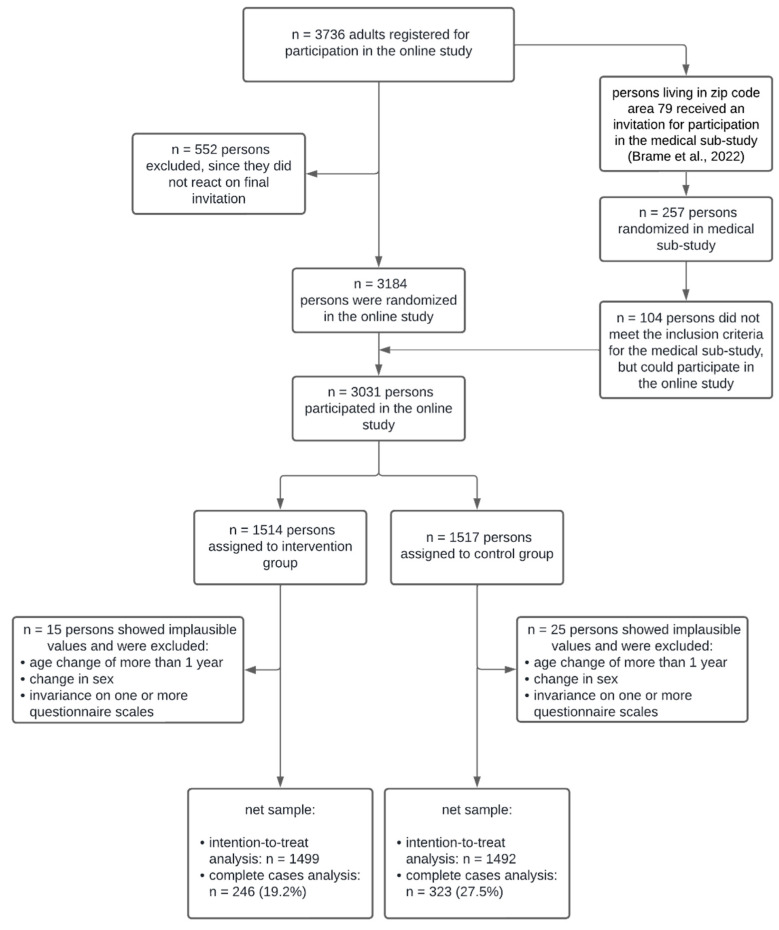
Participant flow in the WLC [42].

After 10 January 2022, we registered 3736 adult persons, who registered for participation in the online study. In total, 552 individuals did not react to the final invitation mail. Individuals from the zip code area 79 (Freiburg, Germany and surroundings) were invited to participate in a medical sub-study, which was conducted parallel to the RCT and is not part of the work presented here (for details see study protocol of the sub-study [42]). Those, who did not meet the criteria for the medical sub-study were conveyed back to the sample for the online study, so that the gross sample was 3031 persons with 1514 in the IG and 1517 in the CG. After elimination of implausible cases (age change from time point to time point greater than 1 year, changing sex or missing variance on one or more questionnaire scales), we ended up with a net sample of 1499 in the IG and 1492 in the CG for the intention-to-treat analyses. For complete cases analyses, the sample was reduced to 19.2% in the IC ending up with *n* = 246 and *n* = 323 in the CG (reduction to 27.5% of the sample). Figure 1 illustrates the participant flow.

The sample showed high dropout rates with about 65% missing values in the IG and about 55% missing values in the CG at each time point after baseline. At baseline, only 5% missings occurred in each group. A total of 57% of the participants with missing values in the IG (respectively 40% in CG) dropped out for all time points after baseline measurement (considered as real dropouts). About 25% of the participants in the IG (and 32% in the CG) had missing values for only one or two measurement time points after t0. 

### 3.2. Sample Description

We achieved equally distributed sociodemographic values in both groups with a mean age of 43 years, 80% of the participants being female and about one third of the sample being unmarried, divorced, widowed or living separately. In total, 76% of the sample in the IG (respectively 77% of the sample in the CG) were mainly in an employment status (see Table 1). This indicates that the randomization was successfully implemented. Less than 3% of the participants reported prior experiences with online weight loss coaching programs. A total of 23% (resp. 22% in the CG) stated to have at least one current mental illness diagnosis. One sixth of the sample categorized itself as smoking and every tenth person reported having tried to lose weight earlier.

Regarding the primary outcome (weight loss in % compared to baseline) a similar pattern for both the CG and the IG could be observed from descriptive perspective: At t1, a mean reduction of 2.8% was achieved in the IG. In the CG, the mean reduction was half as strong with 1.5%. The mean reduction increased at t2 to 3.1% in the IG and 2.2% in the CG. At t3, the mean reduction decreased to 2.6% in the IG and 2.0% in the CG. Considering the absolute body weight in kg, the mean changes from time point to time point turned out to be even smaller. In the IG, a mean reduction of 1.3 kg and in the CG, a mean reduction of 2.0 kg was observed from t0 to t3. Differentiated by sex, women lost less weight on average in both groups (1.8 kg) than men (2.1 kg in the IG, 3.3 kg in the CG) which might be related to the higher baseline weight of 101.9 kg vs. 82.6 kg (IG), respectively 99.4 kg vs. 82.7 kg (CG) for men.

Regarding the covariates used for the multi-level models, no noticeable differences between the two groups were observed. Subjective general health was measured at t0 with 5.3 (resp. 5.2 in CG) in the mean which represents the scale mean of a 11-point scale indicating that respondents rate their general health status equal to others. Health impairment (KOMO) was transformed to a 0 to 10 scale with 0 indicating no comorbidity and 10 high comorbidity. Here, low levels of 0.3 of health impairment by comorbidities were measured for both groups. Mental disorders (PHQ-2) were measured by two 4-point-Likert-scaled items [33] at each time point. For both groups and for each time point, a mean value of about 1.8 was calculated indicating the occurrence of depressive symptoms on some days in the last 2 weeks. Weight Effective and Ineffective (social) support was measured by 14 4-point-Likert scaled items. In both groups and for each time point a mean value of 2.5 (except 2.4 in t1, IG) was observed, indicating the occurrence of social support in relation to weight management between “sometimes” and “often”.

Lastly, we calculated the distribution of user types in the IG, which we identified by Latent Class Analysis (Wurst et al., forthcoming). Only one third of the population (32.6%) was classified as constant users, whose utilization of the program correspond to the usage as intended by the developers of the intervention. In total, 18.4% of the sample used the system only once and another fifth of the sample used the online coach rarely. A total of 12.8% of the sample used the interactive web platform in the first four weeks, and another 15.6% were considered half-time users (6 weeks).

### 3.3. Univariate Analyses

**Figure 2:**
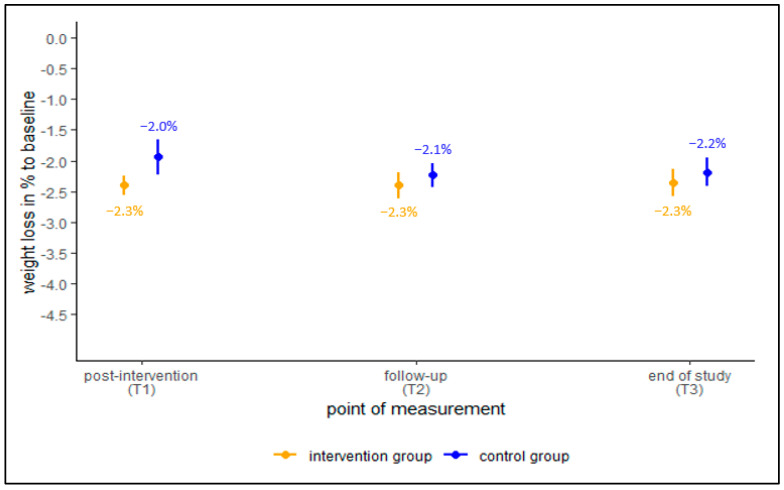
Mean change in weight loss in %, ITT imputed data set, global mean with 95%-confidence intervals.

First, we analyzed the primary outcome “weight loss in % to baseline” over all three time points separated by group. In the ITT data set (Figure 2), members of the control group lost 2.0% of body weight on average at t1 in comparison to baseline, while members of the intervention group lost 2.3% on average. The confidence intervals are not overlapping, indicating that this difference is significant (though small). For the other two time points, the difference in weight loss between the two groups became smaller and was not significant.

**Figure 3:**
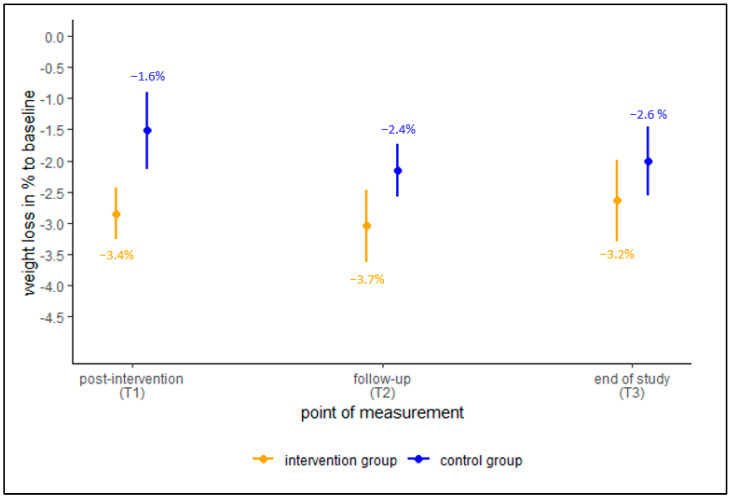
Mean change in weight loss in %, CCA data set, global mean with 95%-confidence intervals.

A similar pattern was observed for the CCA data set (Figure 3). At t1, the mean weight loss in % to baseline was significantly different between the CG (−1.0%) and the IG (−3.4%). Furthermore, the difference was larger than in the ITT comparison. However, at the other two time points, the difference became smaller and insignificant. In general, the confidence intervals in the CCA data set were larger, since the estimation was based on a smaller data set. While the IG’s mean weight loss in % to baseline was stable for each time point in the ITT data set, we found an increase (−3.7%) of weight loss for t2 (in comparison to t1) and a decrease at t3 (−3.2%). Furthermore, we calculated the percentage of those, who managed to reach a weight reduction of more than 5% until t3. One third of the sample in the CCA (32.8%) managed to reach this threshold.

### 3.4. Multi-Level Analyses

First, we estimated intra-class coefficients (ICC) for both the CCA and the ITT based on null models to identify suitability of multilevel modeling. In both cases, the ICC was >0.45 (ITT: 0.93; CCA: 0.92) suggesting high variance predicted by within-cluster effects.

Table 2 shows the results of the multi-level analyses for the ITT (with imputed values) and the CCA data set. The intercept represents the mean body weight at baseline with approx. 95 kg in the ITT and approx. 102 kg in the CCA. The group coefficient can be interpreted as the weight difference between the CG and the IG. Here, being member of the CG was associated with slightly less weight (0.5 kg), though this difference is not statistically significant. For testing of hypothesis H1, the coefficient for time is relevant, indicating that, per each time point difference, a mean weight reduction of 0.4 kg was observed in the ITT and of 0.9 kg in the CCA. This effect was, though small, statistically significant and supports the hypothesis of significant weight loss over time. Hypothesis 2 was tested in this model as well. Here, the interaction term of group and time is important, indicating whether the weight loss effect over time is dependent of the group membership. Since this estimate was not significant, no support for H2 is given and thus, no additional weight reduction in the IG could be found. Furthermore, we identified plausible predictors of weight at t3. Being female and reporting a better self-rated subjective health in relation to others had a significant negative (reductive) effect on body weight, both in the ITT and the CCA data set. Our model identified a significant positive effect of age on body weight (also in both data sets). In the CCA, we found a weight reducing effect of social support and a weight increasing effect of previously diagnosed or present mental disorders. No effect for health impairments by comorbidity was determined.

**Figure 4:**
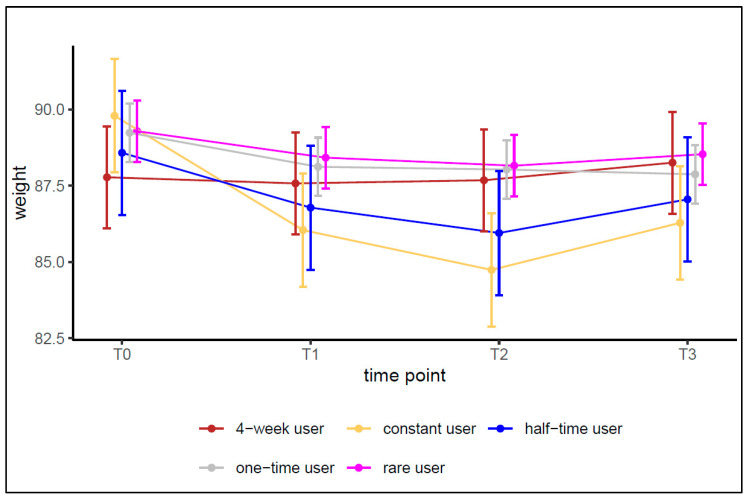
Adjusted weight change by user type, ITT sample.

Lastly, we tested the third hypothesis (H3), whether more intensive use of the coach is associated with higher weight loss. For this purpose, the prior defined user types, found by latent class analysis were used (Wurst et al., forthcoming). The analysis was based on the ITT data set only and adjustments by covariates were made similar to the multi-level models reported in Table 2. The results are graphically displayed in Figure 4. The mean difference in weight between t0 and t1 was strongest for the constant users and became larger comparing t2 with t0. Here, the confidence intervals were not overlapping, indicating a significant weight loss for constant users between t0 and t2. Comparing constant users (considered as more intensive use as the other user types) with the other groups, the confidence intervals were overlapping among the groups for each time point except for t2. Here, a significant difference in weight loss was found between constant users and one-time-users, respectively rare users. Altogether, though constant users showed the lowest mean values in weight for each time point, the differences were not significant (non-overlapping confidence intervals) across groups for t1 or t3, so that no systematic advantage of higher use can be proven. Thus, H3 has to be rejected as well.

### 3.5. Secondary Outcomes

Lastly, we applied our models on the a priori defined secondary outcomes, assuming superiority of the IG in comparison to the CG. We executed the analysis with the ITT data set only (see Table 3). For almost all secondary outcomes, a significant time effect was observed. Physical activity increased approximately about 14 min per week per time point. The other scales also changed in the expected direction. For health-related quality of life, no significant time effect was found. To test whether the IG has shown superior increase/decrease in the secondary outcomes in comparison to the CG, the interaction term of group and time is relevant. Here, no significant coefficient was found, so that we cannot conclude a systematic advantage of the IG in comparison to the CG regarding the secondary outcomes.

## 4. Discussion

Overall, we found weak weight reducing superiority of the intervention against the control group. One argument that might be adduced here is that the possibly good evidence based non-interactive information is as sufficient as complex interactive programs for small weight reducing effects. Both groups contained a large collection of informative material to achieve weight loss, while the IG included interactive components with personalized coaching on algorithm level. However, since both groups showed weak effectiveness in sustainable weight reduction, neither the passive weight loss coaching nor the interactive components are sufficient for long-term weight loss. While we found a significant difference in the weight reducing effect for t1, immediately after completion of the WLC, subjects seem not to be able to implement the behavioral change in a long-term perspective. Thus, further techniques need to be implemented to reach higher sustainability of the weight reducing effects. However, our results do not deviate tremendously from those of other comparable studies, that found a weight loss difference immediately after the study period, but not in the long term perspective [16,43].

A further point that needs to be discussed is whether the found decrease in weight loss is of clinical relevance. We found a mean reduction in body weight of 0.4 kg from time point to time point, which would sum up to less than 2 kg over a time span of 1.3 years. In this context, the cut-off value of 5% loss of body weight is repeatedly mentioned in the literature [16,21,44]. In our CCA data set, 32.8% of the participants could reach a weight reduction of more than 5% until t3. This result appears consistent with the proportion of individuals achieving a weight loss of ≥5% reported in other studies [16,45].

In general, we also have to assume that the body weight measurement is of low validity since it is self-reported and measured by common household scales. This could explain higher weight reducing effects reported in the medical sub-study, where body weight was measured in a laboratory setting under professional supervision (Kohl et al., forthcoming).

A possible explanation for the lower effectiveness in the online study in comparison to the medical sub-study might be a higher motivation of the sub-study population by extrinsic incentivization through regular check-ups. Thus, one could argue that the personal contact at the medical examinations might have had an additional unobserved weight-reducing effect. 

Another potential explanation for the low effects we found might be the composition of the sample. Since the WLC is directed towards the general population, we included not only individuals that had a body mass index of 25 or higher (indicating overweight or adiposities). In the ITT data set, 13.5% of the sample had a BMI up to 25 and 44.2% had a BMI between 25 and 30 at time of inclusion. Thus, 42.3% of the sample were considered obese at t0. Other comparable studies (as well as the medical sub-study), that found larger body weight reducing effects, limited their sample to subjects with abdominal obesity (waist circumference > 102 for men and >88 for women) [46] or BMI  ≥  25 [14,21,43,47], respectively BMI ≥ 27 [16]. This might also explain the higher effects in the medical sub study, since a BMI of 27.5 to 34.9 kg/m^2^ was an inclusion criterion there (Kohl et al., forthcoming).

One aspect that limits the results of our study is the quality of missing data imputation. We conducted sensitivity analyses to quantify the deviance of the estimates and standard errors across the different models (see Table S1, Supplementary Material). In general, the imputed model estimates higher regression coefficients than the ITT model without imputed data (60%) or the CCA model (56%). The standard errors are higher in comparison to the ITT model without imputed data) and lower in comparison to the CCA model. The extent of misestimation is critical and can be differentiated on predictor level (see Table S2, Supplementary Material). Here, some variables show explicitly high levels of misestimation (>55%), e.g., health impairments, the interaction term between group and time or mental disorders. Altogether, it must be assumed that the imputation has not performed well due to the high dropouts over time. For this reason, the CCA model can be considered as fitting better to the real effects; however, CCA generally overestimates coefficients due to the selective sample. Nevertheless, the interpretation of results remains the same, regardless of what model is used.

Furthermore, the assumption of missing at random might be violated as well. It seems plausible, that dropping out might be associated with the intervention itself, e.g., through weak usability or high effort. Follow-up questionnaires were implemented, where possible, to gather more details on reasons for dropping out. In sum, 11% of the subjects enrolled in the WLC could be identified as “active dropouts” who stopped their participation by selecting a dropout button in the online system. We found a slightly higher proportion of those with a BMI ≥ 30 kg/m^2^ in those who were dropping out than in the baseline measurement. We further asked for reasons for dropping out, which could be clustered in personal reasons, technical reasons and reasons that were associated with the online coaching itself. Here, about half of the answers referred to personal reasons and 40% to reasons related to the online coaching. About 10% of all nominations referred to technical problems.

Another effect that could not be controlled but might have influenced the results of this study was the COVID-19 pandemic. Within legal regulations, restricted access to facilities for physical indoor activities might have influenced the behaviors of the subjects in terms of physical exercise. Due to the complex dynamic of the pandemic progress, we were not able to include potential pandemic effects in our models.

Besides the pandemic, other seasonal effects might have occurred. For weight reducing nutrition, high food intake of nutrients with low energy density such as fruits and vegetables is important. These are less available and more expensive in the winter months, which covered the measurement time point t2 for many of our subjects. However, since this circumstance, as well as the pandemic would have affected both the IG and the CG, it would only explain a general lower weight loss effect for both groups and no group difference effect.

Our study also shows some strengths as it covers a large sample size across the German population, while methodologically comparable studies used substantial smaller sample sizes [14,43,47]. Furthermore, the study design as RCT is considered as highest level of evidence [48] combining modern analyses techniques such as multilevel modeling and multiple imputation. Additionally, our study contributes to the body of literature by investigating long-term effectiveness over a larger time span than other studies, that focused on observation periods of 12 to 24 weeks [14,21].

We further tested the coach regarding age responsiveness. There is evidence that weight reduction operates differently across age groups [49]. Therefore, we conducted sensitivity analyses, modeling age as a moderator for the time effect in our MLM models for the IG to test whether the weight loss coaching has an age-dependent effect (see Table S3, Supplementary Material). We found neither for the ITT (not imputed) nor for the CCA data set a significant interaction effect, indicating no conditional effect of age on weight change. Since the WLC was not designed for a specific age group, age-specific analyses were not part of the analysis plan. However, we recommend considering possible age-specific patterns for future development strategies of weight loss interventions in order to examine age-specific effectiveness.

## 5. Conclusions

Participants of the web-based weight loss program could effectively lose weight within 12 weeks after the start of the program. However, we found no sustainable superiority of the interactive component or of more intensive use of the WLC from a long-term perspective. Since the WLC evaluated here targets the general healthy population aiming to reduce and maintain body weight and not explicitly overweight and obese people with (highly) increased BMI, the evaluation was also based on a sample that represents the distribution of body weight within the normal population. Therefore, smaller effects than compared to other studies, including the medical sub-study (Kohl et al., forthcoming), addressing only people with overweight and obesity are not surprising.

## Figures and Tables

**Table 1:** Sample characteristics (intention-to-treat sample).

	Intervention Group				Control Group			
	t0	t1	t2	t3	t0	t1	t2	t3
	**Sample characteristics**							
Age (years) (*M*, [*SD*])	43.0 [13.6]				42.7 [13.9]			
Sex (%)								
female	80.1				79.5			
male	19.6				20.3			
diverse	0.3				0.3			
Family status (%)								
unmarried/divorced/widowed/living sep.	28.3				26.6			
married/in steady partnership	71.7				73.4			
Main activity: employed (%)	76.1				76.9			
Prior experience with online coachings (yes, %)	2.5				2.8			
Earlier/actual diagnosis: mental illness (yes, %)	22.9				22.3			
Smoker (yes, %)	14.5				16.3			
Earlier weight loss tries (no, %)	10.1				12.9			
BMI (%)								
<25	13.5				13.5			
25–30	43.3				45.2			
>30	43.2				41.3			
	**Primary Outcome**							
weight loss in % compared to baseline (*M*, [*SD*])		2.8 [4.5]	3.1 [6.5]	2.6 [7.2]		1.5 [8.1]	2.2 [5.5]	2.0 [6.8]
	**Secondary Outcome**							
Absolute body weight (in kg) (M, [SD])	86.4 [16.1]	84.4 [15.4]	84.0 [16.4]	85.1 [17.5]	86.1 [15.7]	84.2 [15.6]	83.9 [14.9]	84.1 [15.2]
women	82.6 (13.2]	81.3 [13.5]	80.5 [13.8]	80.8 [14.5]	82.7 [13.9]	81.4 [14.6]	80.7 [13.4]	80.9 [13.8]
men *	101.9 [17.4]	97.0 [16.3]	98.4 [17.6]	99.8 [18.8]	99.4 [15.1]	95.0 [14.7]	96.0 [14.2]	96.1 [14.1]
	**Covariates**							
Compared general health status (*M*, [*SD*])	5.3 [2.1]				5.2 [2.1]			
Health impairment (KOMO) (*M*, [*SD*])	0.3 [0.4]				0.3 [0.4]			
Mental disorders (PHQ-2) (*M*, [*SD*])	1.8 [0.7]	1.7 [0.7]	1.8 [0.7]	1.8 [0.7]	1.8 [0.6]	1.8 [0.7]	1.8 [0.7]	1.8 [0.7]
Social Support (WEISS) (*M*, [*SD*])	2.5 [0.5]	2.4 [0.5]	2.4 [0.5]	2.5 [0.5]	2.5 [0.5]	2.5 [0.5]	2.5 [0.5]	2.5 [0.5]
User type (%)								
Constant user	32.6							
Half-time user	15.6							
4-week user	12.8							
One-time user	18.4							
Rare user	20.5							

* due to small sample size, the category “diverse” is not displayed. Note: M = arithmetic mean, SD = standard deviation. Empty cells: was not measured at this time point. Scale range: General health status 0 “worse than others” to 10 “better than others”; KOMO 0 “no comorbidity” to 10 “high comorbidity”; PHQ-2 1 “not at all” to 4 “nearly every day”; WEISS 1 “(almost) never” to 4 “(almost) always”.

**Table 2:** Multi-level analyses on primary outcome (weight in kg).

	Primary Outcome			
	Weight (kg) ITT		Weight (kg) CCA	
**Fixed effects**				
Intercept	**95.13**	(0.94)	**101.71**	(2.62)
group (CG)	−0.47	(0.52)	−0.45	(1.11)
time	**−0.39**	(0.19)	**−0.92**	(0.12)
age	**0.10**	(0.02)	**0.21**	(0.05)
sex (female)	**−8.86**	(0.53)	**−15.24**	(1.34)
subjective general health	**−1.17**	(0.13)	**−2.33**	(0.32)
mental disorder (centered)	0.19	(0.44)	**0.91**	(0.22)
social support (centered)	−0.53	(0.75)	**−1.09**	(0.37)
health impairments	−0.07	(0.68)	−0.73	(1.81)
group × time	−0.03	(0.25)	0.10	(0.16)
**Random effects**				
Intercept	52.64		17.51	
Residual	178.71		158.90	

Note: Unstandardized regression coefficients with standard errors in brackets. Bold values are statistically significant with *p* < 0.05.

**Table 3:** Multi-level analyses on secondary outcomes.

Secondary Outcomes														
	Physical Activity (min/Week)		Eating Behavior		Health-Related Quality of Life		Motivational Self-Efficacy		Volitional Self-Efficacy		Goal Intention		Consequence Expectancy	
**Fixed effects**														
Intercept	**198.27**	(15.88)	**2.56**	(0.03)	**43.31**	(0.51)	**4.45**	(0.06)	**4.45**	(0.06)	**4.25**	(0.06)	**2.96**	(0.02)
group (CG)	−2.45	(8.79)	0.00	(0.02)	−0.06	(0.27)	−0.01	(0.04)	−0.01	(0.04)	−0.02	(0.03)	0.01	(0.01)
time	**13.93**	(3.09)	**0.06**	(0.01)	0.10	(0.11)	**−0.08**	(0.01)	**−0.08**	(0.01)	**−0.07**	(0.01)	0.00	(0.01)
age	−0.10	(0.27)	**0.00**	(0.00)	0.00	(0.01)	0.00	(0.00)	0.00	(0.00)	**0.00**	(0.00)	**0.00**	(0.00)
sex (female)	−2.73	(8.44)	**0.09**	(0.02)	**−0.10**	(0.25)	0.01	(0.03)	0.01	(0.03)	**0.11**	(0.03)	**0.03**	(0.01)
subjective general health	**10.56**	(1.77)	**0.03**	(0.00)	**0.58**	(0.06)	**0.03**	(0.01)	**0.03**	(0.01)	0.00	(0.01)	**0.01**	(0.00)
mental health (centered)	−11.01	(8.78)	**−0.04**	(0.02)	**−2.74**	(0.27)	**−0.08**	(0.03)	**−0.08**	(0.03)	−0.02	(0.03)	**−0.02**	(0.01)
social support (centered)	5.90	(14.08)	**0.07**	(0.03)	**1.28**	(0.41)	**0.40**	(0.06)	**0.40**	(0.06)	**0.23**	(0.05)	**0.09**	(0.02)
health impairments	8.59	(10.04)	0.02	(0.02)	**−5.37**	(0.29)	−0.04	(0.04)	−0.04	(0.04)	0.04	(0.03)	−0.03	(0.02)
group × time	3.83	(4.40)	0.01	(0.01)	0.08	(0.16)	0.00	(0.00)	0.00	(0.02)	−0.01	(0.02)	0.00	(0.01)
**Random effects**														
Intercept	8753.03		0.05		7.58		0.17		0.13		0.08		0.03	
Residual	53,811.73		0.23		56.66		0.98		0.90		0.67		0.10	

Note: Unstandardised regression coefficients with standard errors in brackets. Bold values are statistically significant with *p* < 0.05.

## Data Availability

Not applicable. Data cannot be published due to German Data Protection Law.

## Supplementary Materials

The following supporting information can be downloaded at: https://www.mdpi.com/article/10.3390/ijerph192215157/s1.

## References

[1] Marti A., Moreno-Aliaga M.J., Hebebrand J., Martínez J.A. (2004). Genes, Lifestyles and Obesity. Int. J. Obes..

[2] World Health Organization (2021). Obesity and Overweight.

[3] Schienkiewitz A., Mensink G., Kuhnert R., Lange C. (2017). Übergewicht und Adipositas bei Erwachsenen in Deutschland. J. Health Monit..

[4] Flegal K.M., Kit B.K., Orpana H., Graubard B.I. (2013). Association of All-Cause Mortality with Overweight and Obesity Using Standard Body Mass Index Categories: A Systematic Review and Meta-Analysis. JAMA.

[5] World Cancer Research Fund International (2018). Diet, Nutrition, Physical Activity and Cancer: A Global Perspective: A Summary of the Third Expert Report.

[6] Tremmel M., Gerdtham U.-G., Nilsson P., Saha S. (2017). Economic Burden of Obesity: A Systematic Literature Review. Int. J. Environ. Res. Public Health.

[7] Kent S., Fusco F., Gray A., Jebb S.A., Cairns B.J., Mihaylova B. (2017). Body Mass Index and Healthcare Costs: A Systematic Literature Review of Individual Participant Data Studies: BMI and Healthcare Costs. Obes. Rev..

[8] Die Bundesregierung (2020). Deutsche Nachhaltigkeitsstrategie—Weiterentwicklung 2021.

[9] World Health Organization (2013). Global Action Plan for the Prevention and Control of Noncommunicable Diseases 2013–2020.

[10] Castelnuovo G., Pietrabissa G., Manzoni G.M., Corti S., Ceccarini M., Borrello M., Giusti E.M., Novelli M., Cattivelli R., Middleton N.A. (2015). Chronic Care Management of Globesity: Promoting Healthier Lifestyles in Traditional and MHealth Based Settings. Front. Psychol..

[11] Lara J., Evans E.H., O’Brien N., Moynihan P.J., Meyer T.D., Adamson A.J., Errington L., Sniehotta F.F., White M., Mathers J.C. (2014). Association of Behaviour Change Techniques with Effectiveness of Dietary Interventions among Adults of Retirement Age: A Systematic Review and Meta-Analysis of Randomised Controlled Trials. BMC Med..

[12] Mauro M., Taylor V., Wharton S., Sharma A.M. (2008). Barriers to Obesity Treatment. Eur. J. Intern. Med..

[13] Mensorio M.S., Cebolla-Martí A., Rodilla E., Palomar G., Lisón J.F., Botella C., Fernández-Aranda F., Jimenez-Murcia S., Baños R.M. (2019). Analysis of the Efficacy of an Internet-Based Self-Administered Intervention (“Living Better”) to Promote Healthy Habits in a Population with Obesity and Hypertension: An Exploratory Randomized Controlled Trial. Int. J. Med. Inform..

[14] Mehring M., Haag M., Linde K., Wagenpfeil S., Frensch F., Blome J., Schneider A. (2013). Effects of a General Practice Guided Web-Based Weight Reduction Program—Results of a Cluster-Randomized Controlled Trial. BMC Fam. Pract..

[15] Sorgente A., Pietrabissa G., Manzoni G.M., Rethlefsen M., Simpson S., Perona S., Rossi A., Cattivelli R., Innamorati M., Jackson J.B. (2017). Web-Based Interventions for Weight Loss or Weight Loss Maintenance in Overweight and Obese People: A Systematic Review of Systematic Reviews. J. Med. Internet Res..

[16] Thomas J.G., Raynor H.A., Bond D.S., Luke A.K., Cardoso C.C., Foster G.D., Wing R.R. (2017). Weight Loss in Weight Watchers Online with and without an Activity Tracking Device Compared to Control: A Randomized Trial: Weight Watchers Online. Obesity.

[17] Galani C., Schneider H. (2007). Prevention and Treatment of Obesity with Lifestyle Interventions: Review and Meta-Analysis. Int. J. Public Health.

[18] Fiedler J., Eckert T., Wunsch K., Woll A. (2020). Key Facets to Build up EHealth and MHealth Interventions to Enhance Physical Activity, Sedentary Behavior and Nutrition in Healthy Subjects—An Umbrella Review. BMC Public Health.

[19] Norman G.J., Zabinski M.F., Adams M.A., Rosenberg D.E., Yaroch A.L., Atienza A.A. (2007). A Review of EHealth Interventions for Physical Activity and Dietary Behavior Change. Am. J. Prev. Med..

[20] Vandelanotte C., Müller A.M., Short C.E., Hingle M., Nathan N., Williams S.L., Lopez M.L., Parekh S., Maher C.A. (2016). Past, Present, and Future of EHealth and MHealth Research to Improve Physical Activity and Dietary Behaviors. J. Nutr. Educ. Behav..

[21] Beleigoli A., Andrade A.Q., Diniz M.D.F., Ribeiro A.L. (2020). Personalized Web-Based Weight Loss Behavior Change Program With and Without Dietitian Online Coaching for Adults With Overweight and Obesity: Randomized Controlled Trial. J. Med. Internet Res.

[22] Collins C.E., Morgan P.J., Hutchesson M.J., Callister R. (2013). Efficacy of Standard Versus Enhanced Features in a Web-Based Commercial Weight-Loss Program for Obese Adults, Part 2: Randomized Controlled Trial. J. Med. Internet Res..

[23] Binks M., van Mierlo T. (2010). Utilization Patterns and User Characteristics of an Ad Libitum Internet Weight Loss Program. J. Med. Internet Res..

[24] Hunter C.M., Peterson A.L., Alvarez L.M., Poston W.C., Brundige A.R., Haddock C.K., Van Brunt D.L., Foreyt J.P. (2008). Weight Management Using the Internet. Am. J. Prev. Med..

[25] Franz M.J., Boucher J.L., Rutten-Ramos S., VanWormer J.J. (2015). Lifestyle Weight-Loss Intervention Outcomes in Overweight and Obese Adults with Type 2 Diabetes: A Systematic Review and Meta-Analysis of Randomized Clinical Trials. J. Acad. Nutr. Diet..

[26] Varkevisser R.D.M., van Stralen M.M., Kroeze W., Ket J.C.F., Steenhuis I.H.M. (2019). Determinants of Weight Loss Maintenance: A Systematic Review: Determinants of Weight Loss Maintenance. Obes. Rev..

[27] Moore G.F., Audrey S., Barker M., Bond L., Bonell C., Hardeman W., Moore L., O’Cathain A., Tinati T., Wight D. (2015). Process Evaluation of Complex Interventions: Medical Research Council Guidance. BMJ.

[28] Hariton E., Locascio J.J. (2018). Randomised Controlled Trials—The Gold Standard for Effectiveness Research: Study Design: Randomised Controlled Trials. BJOG Int. J. Obstet. Gynaecol..

[29] McCoy E. (2017). Understanding the Intention-to-Treat Principle in Randomized Controlled Trials. WestJEM.

[30] Shah P.B. (2011). Intention-to-Treat and per-Protocol Analysis. Can. Med. Assoc. J..

[31] Renner B., Hahn A., Schwarzer R., Renner B., Hahn A., Schwarzer R., Freie Universität Berlin (1996). Risiko und Gesundheitsverhalten: Dokumentation der Meßinstrumente des Forschungsprojekts “Berlin Risk Appraisal and Health Motivation Study” (BRAHMS).

[32] Glattacker M., Meixner K., Farin E., Jäckel W.H. (2007). Entwicklung Eines Rehabilitationsspezifischen Komorbiditätsscores Und Erste Prüfung Methodischer Gütekriterien. Phys. Med. Rehabil. Kurortmed..

[33] Kroenke K., Spitzer R.L., Williams J.B.W. (2003). The Patient Health Questionnaire-2 Validity of a Two-Item Depression Screener. Med. Care.

[34] Rieger E., Sellbom M., Murray K., Caterson I. (2018). Measuring Social Support for Healthy Eating and Physical Activity in Obesity. Br. J. Health Psychol..

[35] Fuchs R., Klaperski S., Gerber M., Seelig H. (2015). Messung der Bewegungs- und Sportaktivität mit dem BSA-Fragebogen: Eine methodische Zwischenbilanz. Z. Für Gesundh..

[36] Benyamini Y., Johnston M., Karademas E.C. (2016). Assessment in Health Psychology.

[37] Wurst R., Brame J., Ramsenthaler C., König D., Fuchs R. (2022). A Questionnaire to Assess Eating Behavior: Structure, Validity and Responsiveness of a New German Eating Behavior Scale (SEV). Appetite.

[38] Ochsner S., Scholz U., Hornung R. (2013). Testing Phase-Specific Self-Efficacy Beliefs in the Context of Dietary Behaviour Change: Phase-Specific Self-Efficacy Beliefs. Appl. Psychol. Health Well-Being.

[39] Bullinger M., Kirchberger I. (1998). SF-36 Fragebogen Zum Gesundheitszustand.

[40] Tinsel I., Metzner G., Schlett C., Sehlbrede M., Bischoff M., Anger R., Brame J., König D., Wurst R., Fuchs R. (2021). Effectiveness of an Interactive Web-Based Health Program for Adults: A Study Protocol for Three Concurrent Controlled-Randomized Trials (EVA-TK-Coach). Trials.

[41] Resche-Rigon M., White I.R. (2018). Multiple Imputation by Chained Equations for Systematically and Sporadically Missing Multilevel Data. Stat. Methods Med. Res..

[42] Brame J., Kohl J., Wurst R., Fuchs R., Tinsel I., Maiwald P., Fichtner U., Armbruster C., Bischoff M., Farin-Glattacker E. (2022). Health Effects of a 12-Week Web-Based Lifestyle Intervention for Physically Inactive and Overweight or Obese Adults: Study Protocol of Two Randomized Controlled Clinical Trials. Int. J. Environ. Res. Public Health.

[43] Patrick K., Calfas K.J., Norman G.J., Rosenberg D., Zabinski M.F., Sallis J.F., Rock C.L., Dillon L.W. (2011). Outcomes of a 12-Month Web-Based Intervention for Overweight and Obese Men. Ann. Behav. Med..

[44] Horn D.B., Almandoz J.P., Look M. (2022). What Is Clinically Relevant Weight Loss for Your Patients and How Can It Be Achieved? A Narrative Review. Postgrad. Med..

[45] Gudzune K.A., Doshi R.S., Mehta A.K., Chaudhry Z.W., Jacobs D.K., Vakil R.M., Lee C.J., Bleich S.N., Clark J.M. (2015). Efficacy of Commercial Weight-Loss Programs: An Updated Systematic Review. Ann. Intern. Med..

[46] Hansel B., Giral P., Gambotti L., Lafourcade A., Peres G., Filipecki C., Kadouch D., Hartemann A., Oppert J.-M., Bruckert E. (2017). A Fully Automated Web-Based Program Improves Lifestyle Habits and HbA1c in Patients With Type 2 Diabetes and Abdominal Obesity: Randomized Trial of Patient E-Coaching Nutritional Support (The ANODE Study). J. Med. Internet Res..

[47] Grey E.B., Thompson D., Gillison F.B. (2019). Effects of a Web-Based, Evolutionary Mismatch-Framed Intervention Targeting Physical Activity and Diet: A Randomised Controlled Trial. Int. J. Behav. Med..

[48] Burns P.B., Rohrich R.J., Chung K.C. (2011). The Levels of Evidence and Their Role in Evidence-Based Medicine. Plast. Reconstr. Surg..

[49] Svetkey L.P., Clark J.M., Funk K., Corsino L., Batch B.C., Hollis J.F., Appel L.J., Brantley P.J., Loria C.M., Champagne C.M. (2014). Greater Weight Loss with Increasing Age in the Weight Loss Maintenance Trial. Obesity.

