# Supplementary figures and images for: Evaluation of an Interactive Web-Based Health Program for Weight Loss—A Randomized Controlled Trial
Source: Int J Environ Res Public Health. 2022 Nov 17;19(22):15157. doi: 10.3390/ijerph192215157 (PMC9690889; doi:10.3390/ijerph192215157)

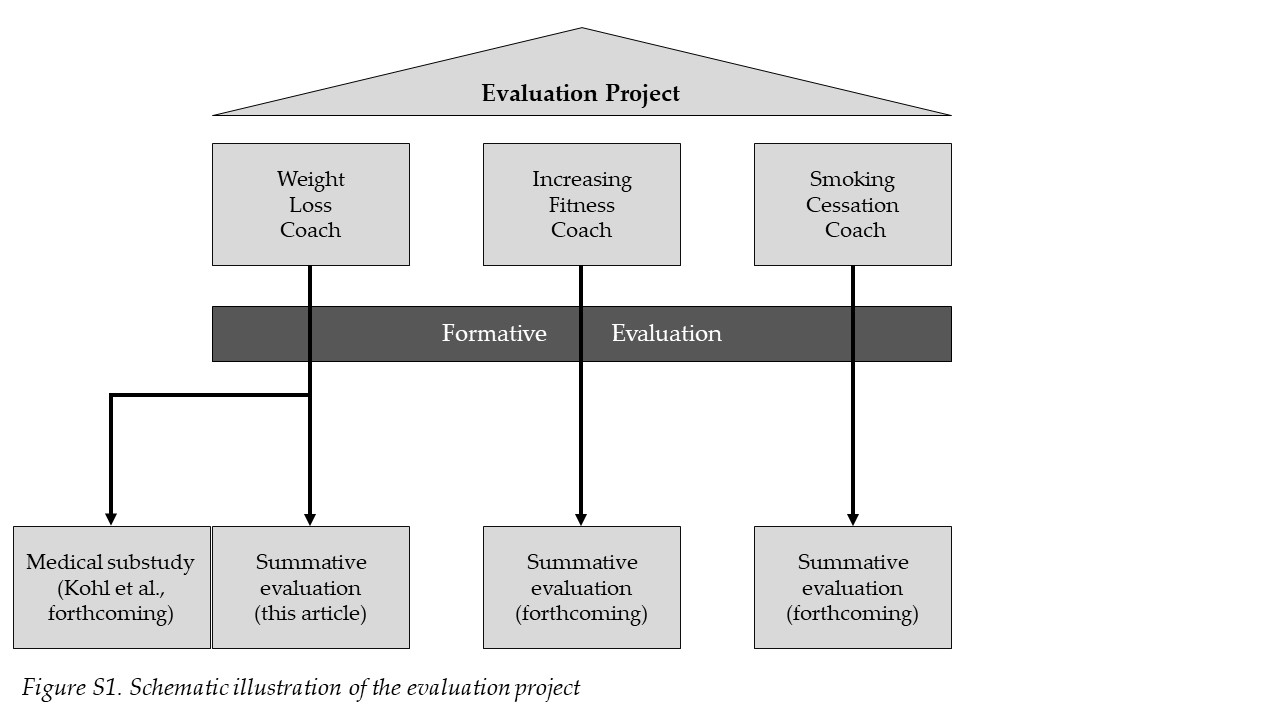

Supplement: Supplementary file 1 [file ijerph-19-15157-s001.zip › FigureS1.jpg]
